# Pure SILS Floppy Nissen Fundoplication with Hiatal Repair: A Case Report

**DOI:** 10.5402/2011/347487

**Published:** 2011-07-03

**Authors:** Umut Barbaros, Tugrul Demirel, Aziz Sumer, Ugur Deveci, Mustafa Tukenmez, Mehmet Ibrahim Cansunar, Murat Kalayci, Ahmet Dınccag, Ridvan Seven, Selcuk Mercan

**Affiliations:** ^1^Department of General Surgery, Istanbul Faculty of Medicine, University of Istanbul, Istanbul, Turkey; ^2^Department of General Surgery, Memorial Hizmet Hospital, Istanbul, Turkey; ^3^Department of General Surgery, Faculty of Medicine, Yuzuncuyıl University, Van, Turkey; ^4^Department of General Surgery, Faculty of Medicine, Maltepe University, Istanbul, Turkey; ^5^Department of General Surgery, Canakkale Military Hospital, Canakkale, Turkey; ^6^Department of General Surgery, Faculty of Medicine, Yeditepe University, Canakkale, Turkey

## Abstract

*Background*. Single-incision laparoscopic surgery has recently became popular on behalf of inventing less invasive procedures. In this paper, we present a case of Pure SILS Nissen Fundoplication. *Patient and Methods*. In February 2010 a 29-year old male patient with a 4 cm sliding hiatus hernia presenting with reflux symptoms had undergone a standard floppy Nissen Fundoplication with a hiatus repair via single 2 cm incision in umbilicus. *Results*. The procedure had obeyed the standard natural orifice surgery rules, and no needlescopic assistance for any stage of the operation was used so to be a pure single-incision procedure. The operation lasted for 120 minutes without any need of conversion, and the patient was discharged the following day of operation. *Conclusion*. In the recent time, hybrid single incision laparoscopy techniques have been defined with the use of extra-abdominal supplements for retraction of liver or stomach for Nissen procedure. In addition the main issue in single-incision upper GI and/or hiatus surgery is still the retraction of liver. We succeeded to retract the left lobe of liver through the incision and completed the operation without any need for supplemental access besides the umbilical incision till the end. SILS Hiatus Surgery can be safely and effectively done but the issue needs further clinical studies to state the efficacy when compared to standard laparoscopy.

## 1. Introduction

In the last 30 years, laparoscopic procedures have been used in various fields for treatment of many malignant and benign diseases. Currently, laparoscopy is preferred as an alternative to standard surgery due to its low complication rates, less postoperative pain, achievement of better cosmetic results, and early recovery of patients returning them to daily life faster. Surgery of gastroesophageal reflux disease (GERD) either alone or accompanied by any stage and/or type of hiatus hernia had also entered a new era by the introduction of video-endoscopic techniques in the last three decades. Dallemagne et al. reported the first laparoscopic Nissen Fundoplications in 1991 [1], and since then the laparoscopic Nissen Fundoplication has been accepted as the gold standard therapy for GERD. 

As the efforts aiming to reduce incision of laparoscopic surgery for better cosmetic appearance have been the main issue, investigators have begun to perform the endoscopic procedures via natural orifices instead of using the conventional laparoscopic techniques [2]. However, this technology necessitates a visceral injury. For this purpose safer and equally as cosmetic approach use of single-incision laparoscopic surgery (SILS) has gained significant momentum in different fields of surgical practice [3–5]. In our clinic, the first transvaginal cholecystectomy practices (NOTES: natural orifice transluminal endoscopic surgery) have been conducted by Seven and Barbaros [6]. Moreover, SILS practice has been started in our clinic since January 2009. Single-incision laparoscopic splenectomy (SILSp) technique has been first described by Barbaros and Dinççağ [7].

In this paper we are reporting the pure SILS Nissen Fundoplication. 

## 2. Materials and Methods

A 29-year old male patient presenting with reflux symptoms and a 4 cm type I hiatus hernia had undergone a standard floppy Nissen Fundoplication with a hiatus repair via single 2 cm incision in umbilicus. The operation duration was 120 minutes from first skin incision to application of wound dresses, and the procedure was completed totally via special SILS Port by Covidien. The patient received non steroid anti-inflammatory agent (8 mg intravenous lornoxicam, twice a day) and narcotic analgesic (50 mg intramuscular pethidine, twice a day) and was discharged the day after the operation. 

### 2.1. Technique

The procedure was performed using SILS instruments, and the intervention was conducted via special SILS port (Covidien, Mansfield, MA, USA), specifically designed for SILS. A skin incision of 2 cm was made through umbilicus, and the SILS Port was introduced by a Sims-Mayer towel clamp to the abdominal cavity. After obtaining a 14 mmHg pneumoperitoneum, a 5 mm camera port was introduced and the abdominal cavity was explored. Two additional 5 mm ports were introduced under laparoscopic vision. After further hiatus exploration, the insufflation cannula was extracted and replaced by a standard 5 mm port with a Luer Lock connection for insufflation. A 5 mm Snowden Pencer liver retractor was introduced through this latter port and placed under liver to retract the left lobe towards the abdominal wall. The rest of the procedure was identical to the standard Floppy Nissen Fundoplication. The dissection started with the division of Leimer ligament. The right crura was retracted laterally with a roticulated grasper, and peritoneum was incised with electrocautery hook. The hernia sac was dissected and totally mobilized by using 5 mm LigaSure device of Covidien in a standard fashion from right to left. Esophagus was further mobilized in the thorax, and the gastrosplenic ligament was divided by using 5 mm Ligasure. After completion of dissection and removal of the whole hernia sac, the left-sided 5 cmm cannula was extracted and replaced by a 12 mm one to introduce Endo Stitch suturing device of Covidien. By retracting the esophagus with the roticulated grasper upwards two stitches of 00 silk were placed by Endo-stitch suturing device. The fundus was passed through the retro esophageal sulcus and fundoplication of 2-3 cm was matured using Endo Stitch suturing device by placing two 00 silk sutures. No drain was placed, and the port site was repaired with thick nonabsorbable suture. Skin incision was sutured with 4/0 absorbable monofilament suture material. 

## 3. Results

A 29-year old male patient presenting with reflux symptoms and type l hiatus hernia was operated through a 2 cm umbilical incision via SILS port. After undergoing a standard floppy Nissen Fundoplication, the patient was discharged without any complications. 

## 4. Discussion

The introduction of laparoscopy in the early 1990s leaded a new era in the surgical treatment of human diseases. Evolution of minimally invasive techniques has furthered an impulsion in the surgical community to reduce the invasiveness of laparoscopic surgery. Although SILS practice is relatively new, it has already been applied in plenty of surgical procedures by keen on surgeons. Many procedures such as cholecystectomy, adrenalectomy, laparoscopic total extraperitoneal inguinal hernia repair, right hemicolectomy, left hemicolectomy, rectum operations, sleeve gastrectomy, gastrojejunostomy, and nephrectomy have been performed through single-incision and reported to the world literature [8–18]. 

Performing Nissen Fundoplication from a single incision through the umbilicus has perfect cosmetic results. Although it is not yet possible to state that single incision fundoplications have superiority over standard laparoscopic surgery, this preliminary experience has encouraged us to deal with further developments of the technique. The swords fights of the instruments as in all SIL procedures are also of concern in SILS Nissen Fundoplication. The surgeon must be experienced to use the combinations of both the standard and roticulated instruments. The main issue related to the surgery of hiatus is the retraction of the left lobe of the liver. Several techniques were defined for liver retraction in single-incision procedures such as hanging the liver with silastic bands, elastic stays with hook, and we have used infusion lines to hang the liver to anterior abdominal wall by a thick suture passed through the infusion line and taken out of the abdomen with a suture passer in our previous laparoscopic gastric resections. We used the insufflation line in the SILS port of Covidien to place an extra standard 5 mm port with a Luer-Lock mechanism for insufflation. We placed the Snowden Pencer liver retractor through this port, and the insufflation was maintained via this port. Using the liver retractor through the SILS port did not handicap the use hand instruments in the operative field. 

Use of a long shafted laparoscopic camera is useful as to leave the operator freer space at the operative theatre. We also used 5 mm–42 cm long telescopes in all of the SILS cases. 

In conclusion, we believe SILS is as beneficial as conventional laparoscopic surgery regarding the major advantages of laparoscopy. Although SILS provides a better cosmetic outcome, in order to reveal the main advantages of SILS, more prospective and randomized studies are required. 
